# Acute Renal and Hemodynamic Risk in Young Adults With Sickle-Cell Trait and Disease: A Propensity-Score-Matched Analysis

**DOI:** 10.7759/cureus.117299

**Published:** 2026-10-01

**Authors:** Matthew A Peterman, Jane C Brennan, Andrea H Johnson, James York, Benjamin M Petre, Daniel E Redziniak, Justin Turcotte

**Affiliations:** 1 Orthopedic Research, Luminis Health Anne Arundel Medical Center, Annapolis, USA; 2 Orthopedics, Luminis Health Anne Arundel Medical Center, Annapolis, USA; 3 Orthopedic Surgery, Luminis Health Anne Arundel Medical Center, Annapolis, USA; 4 Orthopedic and Surgical Research, Luminis Health Anne Arundel Medical Center, Annapolis, USA

**Keywords:** acute renal failure, adolescent, heat-related illness, hemodynamic shock, sickle cell disease, sickle cell trait, young adult

## Abstract

Background

Sickle-cell trait (SCT) is historically regarded as a benign carrier state, although emerging evidence from athletic and military cohorts suggests increased vulnerability during physiological stress. In this study, we aimed to identify the incidence of acute renal and hemodynamic complications in adolescent and younger adult patients with SCT or sickle-cell disease (SCD) and compare these risks against propensity-matched controls from the general non-sickle-cell population.

Methods

A retrospective analysis of the TriNetX global network was performed. Patients aged 13-40 years with SCD or SCT were identified and propensity-score matched to non-sickle-cell controls based on age, sex, race, comorbidities, and medication use. Univariate analyses and Kaplan-Meier survival analysis assessed relative risk and time-to-event for rhabdomyolysis, heat-related illness, acute kidney failure, dehydration, electrolyte imbalance, syncope/collapse, and hemodynamic shock within five years. Subgroup analysis of differences in outcomes between SCD and SCT patients was performed.

Results

A total of 24,178 patients remained per cohort (SCD/SCT and no SCD/SCT) after matching. Compared to controls, the sickle-cell cohort exhibited significantly higher risks of dehydration (RR = 1.62, 95% CI 1.48-1.77), acute kidney failure (RR = 1.76, 95% CI 1.56-1.98), electrolyte imbalance (RR = 1.66, 95% CI 1.55-1.78), and hypotension/volume-depletion shock (RR = 1.71, 95% CI 1.47-1.99) (all P < 0.001). In subgroup analysis, SCD showed higher risk than SCT for most outcomes, but they experienced less syncope/collapse (RR = 0.74, 95% CI 0.64-0.85, P < 0.001).

Conclusion

Adolescents and young adults with sickle-cell hemoglobinopathies face significant renal and hemodynamic vulnerabilities. Higher collapse incidence in SCT suggests unique susceptibility to acute hemodynamic failure. These findings have generated hypotheses that are worth prospective investigations, including the development of universal-precaution protocols and monitoring key subclinical biomarkers, which might help identify patients undergoing significant physiological strain before a clinical crisis develops. However, further prospective research incorporating detailed laboratory data and extended observation periods is necessary to determine if such strategies can be effectively translated into evidence-based care for the wider young-adult sickle-cell population.

## Introduction

Sickle-cell trait (SCT) is defined by the heterozygous inheritance of the sickle hemoglobin gene (HbS) and affects approximately 300 million people worldwide [1,2]. Within the United States, the prevalence is estimated at 7% to 9% among the African American population [1,2]. In contrast, sickle-cell disease (SCD) results from the homozygous inheritance of HbS, leading to severe chronic anemia, recurrent vaso-occlusive crises, organ damage, and reduced life expectancy [3-5]. While SCT is typically considered a benign carrier state, often with normal red blood cell indices and life expectancy comparable to the general population, individuals with SCD experience lifelong debilitation and early mortality [2,6]. However, this classification of SCT as a benign state has been increasingly challenged by large-scale studies of military recruits and elite athletes, which reveal a disproportionate incidence of acute, life-threatening events during high-intensity exertion [7-10]. Furthermore, although the condition is often asymptomatic when the HbS blood cell percentage is less than 35%, metabolic crises can be precipitated by physiological stressors such as severe hypoxia, hyperthermia, or dehydration [2,11].

Despite these alarming clinical signals, the evidence base for the acute-care management of SCT remains limited [1,2,12]. Systematic reviews of SCT-related outcomes have established high-strength evidence for only a narrow range of conditions, such as pulmonary embolism, proteinuria, and chronic kidney disease, while most other potential complications lack robust supporting data [1,2,12]. Within the emergency department (ED), current guidelines for acute presentations of SCT patients are often based on “moderately weak” evidence, leaving a significant lack of consensus regarding optimal fluid repletion or pain management strategies [12,13].

Current epidemiological insights are largely derived from specialized cohorts, such as military recruits and elite athletes, which may not accurately reflect the risks faced by the broader young-adult population presenting for routine acute care [1,7,10]. This specialized focus leaves a significant gap in our understanding of how chronic vulnerabilities, such as hyposthenuria and relative systemic hypertension, translate into acute complications in a general hospital setting [11,14,15]. Our focus is the young-adult population, as this group typically includes the majority of individuals who participate in competitive athletics or demanding physical training where the stress of intense exercise may uncover the clinical relevance of SCT [10,16]. To address these deficiencies, this study utilized a global electronic health record database to analyze a cohort of propensity-score-matched individuals with SCD and SCT, controlled for age, sex, race, comorbidities, and medication use. We evaluated the incidence of acute renal failure, hemodynamic shock, and electrolyte imbalance after an index ED or inpatient encounter, comparing patients with sickle-cell hemoglobinopathies to non-sickle-cell controls and comparing SCD versus SCT cohorts.

## Materials and methods

Study population

This study was deemed exempt by the institutional review board as a review of de-identified, aggregated data by the institutional clinical research committee. The TriNetX Research Network (TriNetX) database was queried as of November 18, 2025. Patient cohorts and outcome measures were defined using International Classification of Diseases, 10th Revision (ICD-10) diagnosis codes and Current Procedural Terminology (CPT) codes. Eligible patients were required to have an inpatient or ED encounter between January 1, 2020, and January 1, 2024, and to be between 13 and 40 years old. The date of the inpatient or ED visit served as the index encounter within the study window.

To minimize confounding from other hematologic conditions, patients with the following diagnoses were excluded: thalassemia (ICD-10: D56), nutritional anemias (ICD-10: D50-D53), aplastic and other anemias or bone marrow failure syndromes (ICD-10: D60-D64), neoplasms (ICD-10: C00-D49), anemia due to enzyme disorders (ICD-10: D55), other hereditary hemolytic anemias (ICD-10: D58), and acquired hemolytic anemia (ICD-10: D59). Patients were then divided into two cohorts by whether they had a diagnosis of sickle-cell disorders (SCD or SCT, ICD-10: D57) or not.

Study outcomes

The primary outcome was the occurrence of any of the following adverse events from one day to five years following the index encounter: rhabdomyolysis (ICD-10: M62.82), heat-related illness (ICD-10: T67.0-T67.6), dehydration (ICD-10: E86.0), acute kidney failure (ICD-10: N17), electrolyte imbalance (ICD-10: E87.1-E87.6), syncope or collapse (ICD-10: R55), or hypotension/volume-depletion shock (ICD-10: I95, R57.1). Secondary outcomes were individual rates of the above conditions in patients with SCD or SCT compared to healthy controls.

Statistical analysis

Propensity-score matching was performed on age, sex, race, and the following diagnosis and medication categories: hematologic and immune disorders (ICD-10: D50-D89), musculoskeletal and connective tissue disorders (ICD-10: M00-M99), endocrine and metabolic disorders (ICD-10: E00-E89), cardiovascular disorders (ICD-10: I00-I99), mental and behavioral health disorders (ICD-10: F01-F99), cardiovascular medications (CV000), blood and volume replacement medications (BL000), and central nervous system medications (CN000). The PSM was performed using 1:1 greedy, nearest-neighbor matching with a caliper of 0.1 pooled standard deviations. Cohort demographics and comorbidities were compared using two-sided independent-samples t-tests and chi-square tests. Matched cohort outcomes were compared using risk ratios with 95% confidence intervals.

Kaplan-Meier survival analysis was performed to evaluate time to the first adverse event, and differences between cohorts were assessed using the log-rank test.

A subgroup analysis further separated the sickle-cell cohort into patients with SCD and those with SCT. SCD was defined using the following ICD-10 codes: Hb-SS disease with crisis (D57.0), SCD without crisis (D57.1), sickle-cell/Hb-C disease (D57.2), sickle-cell thalassemia (D57.4), and other sickle-cell disorders (D57.8). SCT was defined as D57.3 and excluded all codes used to define SCD. Outcomes for these subgroups were analyzed using risk ratios with 95% confidence intervals, and time-to-event differences were evaluated using Kaplan-Meier analysis with the log-rank test. Statistical significance was set at p<0.05. All analyses were conducted within the TriNetX platform.

TriNetX

TriNetX is a global research network that includes data from more than 170 healthcare organizations across 30 countries and over 400 million patients [17]. Of the participating organizations, 76 share data for aggregated outcomes research and are included in this study. Variables captured include demographics, medications, lab values, diagnoses mapped to ICD-10 coding, and procedures mapped to CPT coding. Health Insurance Portability and Accountability Act (HIPAA) compliant electronic health record data are collected from participating healthcare organizations that submit structured and unstructured data elements. TriNetX is a federated network and received a waiver from the Western Institutional Review Board, as the only data received include aggregated counts and statistical summaries of de-identified information. No protected health information is exchanged in retrospective analyses.

## Results

Of the 9,291,902 patients included in this study, 24,185 (0.3%) had a diagnosis of sickle-cell disorder. Prior to propensity score matching, patients with sickle-cell disorder were more likely to be female (16,976 (70.2%) vs. 5,101,777 (55.0%); p<0.001) and Black or African American (20,168 (83.4%) vs. 1,868,819 (20.2%); p<0.001). They were also slightly younger than those without sickle-cell disorder (25.9 ± 7.6 vs. 26.8 ± 7.8 years; p<0.001).

The sickle-cell disorder cohort demonstrated substantially higher rates of multiple comorbidity categories, including hematologic and immune disorders (9,118 (37.7%) vs. 225,416 (2.4%); p<0.001), musculoskeletal and connective tissue disorders (8,413 (34.8%) vs. 1,552,590 (16.8%); p<0.001), endocrine and metabolic disorders (6,556 (27.1%) vs. 952,806 (10.3%); p<0.001), cardiovascular disorders (3,865 (16.0%) vs. 482,610 (5.2%); p<0.001), and mental and behavioral health disorders (6,711 (27.7%) vs. 1,409,891 (15.2%); p<0.001). They also had increased utilization of cardiovascular medications (8,382 (34.7%) vs. 1,261,589 (13.6%); p<0.001), blood and volume replacement medications (4,395 (18.2%) vs. 361,379 (3.9%); p<0.001), and central nervous system medications (14,576 (60.3%) vs. 2,656,682 (28.7%); p<0.001) (Table 1).

**Table 1:** Patient Demographics, Diagnoses, and Medications Prior to Matching * P-value < 0.001. All data are presented as n (%) or mean ± standard deviation; continuous variables were compared using independent-samples t-tests, and categorical variables were compared using Pearson χ² tests.

	Sickle Cell (n=24,185)	No Sickle Cell (n=9,267,717)	Test Statistic	P-value
Demographics				
Age, years	25.9 ± 7.6	26.8 ± 7.8	t = -18.39	<0.001*
Female	16,976 (70.2)	5,101,777 (55.0)	χ² = 2,235.84	<0.001*
Black or African American	20,168 (83.4)	1,868,819 (20.2)	χ² = 59,535.93	<0.001*
Diagnoses				
Hematologic & Immune Disorders	9,118 (37.7)	225,416 (2.4)	χ² = 121,954.16	<0.001*
Musculoskeletal & Connective Tissue Disorders	8,413 (34.8)	1,552,590 (16.8)	χ² = 5,612.32	<0.001*
Endocrine & Metabolic Disorders	6,556 (27.1)	952,806 (10.3)	χ² = 7,376.77	<0.001*
Cardiovascular Disorders	3,865 (16.0)	482,610 (5.2)	χ² = 5,643.26	<0.001*
Mental & Behavioral Health Disorders	6,711 (27.7)	1,409,891 (15.2)	χ² = 2,933.62	<0.001*
Medications				
Cardiovascular Medications	8,382 (34.7)	1,261,569 (13.6)	χ² = 9,054.58	<0.001*
Blood & Volume Replacement Medications	4,395 (18.2)	361,379 (3.9)	χ² = 12,995.22	<0.001*
Central Nervous System Medications	14,576 (60.3)	2,656,682 (28.7)	χ² = 11,761.37	<0.001*

After propensity-score matching, 24,178 patients remained in each cohort. There were no significant differences in demographics, diagnoses, or medication utilization between the matched groups (Table 2).

**Table 2:** Patient Demographics, Diagnoses, and Medications After Matching All data are presented as n (%) or mean ± standard deviation; continuous variables were compared using independent-samples t-tests, and categorical variables were compared using Pearson χ² tests.

	Sickle Cell (n=24,178)	No Sickle Cell (n=24,178)	Test Statistic	P-value
Demographics				
Age, years	25.9 ± 7.6	26.0 ± 7.5	t = -1.45	0.062
Female	16,969 (70.2)	17,052 (70.5)	χ² = 0.68	0.409
Black or African American	20,161 (83.4)	20,162 (83.4)	χ² < 0.01	0.990
Diagnoses				
Hematologic & Immune Disorders	9,111 (37.7)	9,110 (37.7)	χ² < 0.01	0.993
Musculoskeletal & Connective Tissue Disorders	8,406 (34.8)	8,423 (34.8)	χ² = 0.03	0.871
Endocrine & Metabolic Disorders	6,550 (27.1)	6,562 (27.1)	χ² = 0.02	0.902
Cardiovascular Disorders	3,859 (16.0)	3,826 (15.8)	χ² = 0.17	0.681
Mental & Behavioral Health Disorders	6,711 (27.8)	6,708 (27.7)	χ² < 0.01	0.976
Medications				
Cardiovascular Medications	8,376 (34.6)	8,354 (34.6)	χ² = 0.04	0.833
Blood & Volume Replacement Medications	4,388 (18.1)	4,494 (18.6)	χ² = 1.55	0.213
Central Nervous System Medications	14,569 (60.3)	14,589 (60.3)	χ² = 0.03	0.853

The average follow-up time was longer for the sickle-cell disorder cohort compared with the matched no sickle-cell cohort (946.6 ± 632.5 vs. 655.1 ± 636.3 days). There were no significant differences in the rate of rhabdomyolysis, heat-related illness, or syncope/collapse. However, patients with sickle-cell disorder were at significantly increased risk for several adverse outcomes, including dehydration (RR: 1.62; 95% CI: 1.48-1.77; p<0.001), acute kidney failure (RR: 1.76; 95% CI: 1.56-1.98; p<0.001), electrolyte imbalance (RR: 1.66; 95% CI: 1.55-1.78; p<0.001), and hypotension/volume-depletion shock (RR: 1.71; 95% CI: 1.47-1.99; p<0.001) (Table 3).

**Table 3:** Outcomes After Matching * P-value < 0.001. All data are presented as n (%) or mean ± standard deviation; continuous variables were compared using independent-samples t-tests, and categorical variables were compared using Pearson χ² tests.

Outcome	Sickle Cell (n=24,178)	No Sickle Cell (n=24,178)	Risk Ratio (Sickle Cell vs. No Sickle Cell)	Test Statistic	P-value
Follow-Up Time (Days)	946.6 ± 632.5	655.1 ± 636.3	N/A	t = 50.52	N/A
Rhabdomyolysis	98 (0.4)	88 (0.4)	1.11 (0.84, 1.48)	χ² = 0.54	0.463
Heat-Related Illness	13 (0.1)	14 (0.1)	0.93 (0.44, 1.98)	χ² = 0.04	0.847
Dehydration	1,156 (4.8)	715 (3.0)	1.62 (1.48, 1.77)	χ² = 108.13	<0.001*
Acute Kidney Failure	711 (2.9)	405 (1.7)	1.76 (1.56, 1.98)	χ² = 85.89	<0.001*
Electrolyte Imbalance	1,993 (8.2)	1,198 (5.0)	1.66 (1.55, 1.78)	χ² = 212.06	<0.001*
Syncope/Collapse	863 (3.6)	805 (3.3)	1.07 (0.98, 1.18)	χ² = 2.09	0.148
Hypotension/Volume-Depletion Shock	442 (1.8)	259 (1.1)	1.71 (1.47, 1.99)	χ² = 48.48	<0.001*

Time-to-event analysis showed a significantly shorter time to any adverse event in the sickle-cell disorder cohort compared with the matched cohort (log-rank p<0.001) (Figure 1).

**Figure 1:**
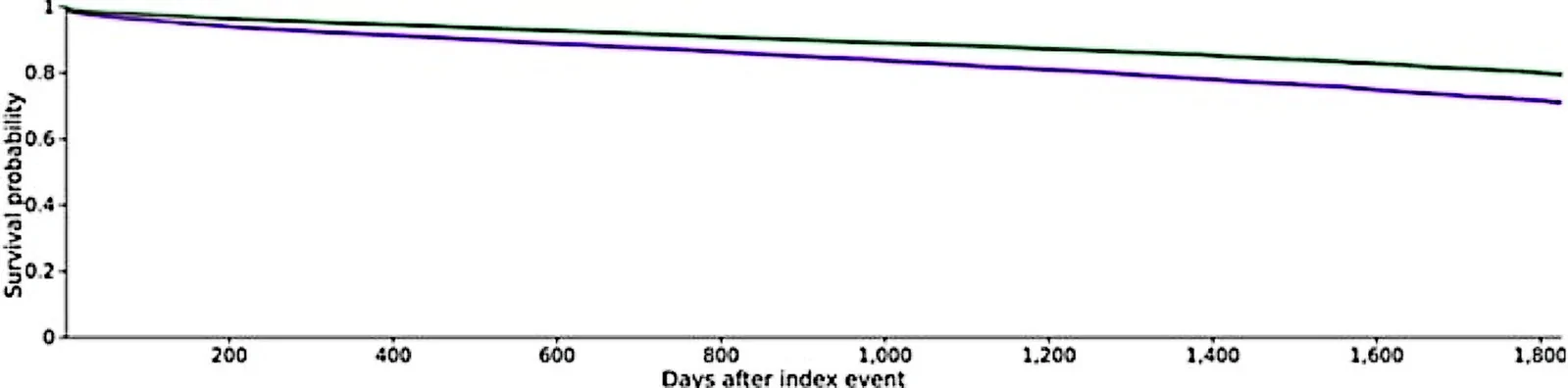
Kaplan-Meier Survival Curve Comparing Time to Adverse Event Comparing Sickle-Cell to No Sickle-Cell Patients Sickle-cell patients = purple line; No sickle-cell patients = green line; log-rank p < 0.001.

In the subgroup analysis, patients with SCD demonstrated substantially higher rates of most adverse outcomes compared with those with SCT. Follow-up time was similar between groups. SCD was associated with significantly increased risks of dehydration (RR: 2.76, 95% CI: 2.45-3.11; p<0.001), acute kidney failure (RR: 2.58, 95% CI: 2.21-3.00; p<0.001), electrolyte imbalance (RR: 2.34, 95% CI: 2.14-2.55; p<0.001), and hypotension or volume-depletion shock (RR: 2.19, 95% CI: 1.81-2.65; p<0.001). Rates of rhabdomyolysis were low and did not differ significantly between groups (RR: 1.34, 95% CI: 0.90-2.00; p=0.151), and heat-related illness was not reportable for either subgroup, as event counts fell below the minimum threshold required for disclosure (<10 events). Notably, syncope/collapse occurred more frequently in individuals with SCT (563 (4.0%) vs. 297 (2.9%)), with SCD demonstrating a lower relative risk (RR: 0.74, 95% CI: 0.64-0.85; p<0.001) (Table 4).

**Table 4:** Subgroup Analysis: Sickle-Cell Outcomes by Disease Status * P-value < 0.001. All data are presented as n (%) or mean ± standard deviation; continuous variables were compared using independent-samples t-tests, and categorical variables were compared using Pearson χ² tests.

Outcome	Sickle-Cell Disease (n=10,092)	Sickle-Cell Trait (n=14,084)	Risk Ratio (Disease vs. Trait)	Test Statistic	P-value
Follow-Up Time (Days)	935.3 ± 677.5	948.7 ± 596.6	N/A	t = -1.34	N/A
Rhabdomyolysis	47 (0.5)	49 (0.3)	1.34 (0.90, 2.00)	χ² = 2.06	0.151
Heat-Related Illness	N/A	N/A	N/A	N/A	N/A
Dehydration	766 (7.6)	387 (2.7)	2.76 (2.45, 3.11)	χ² = 212.9	<0.001*
Acute Kidney Failure	460 (4.6)	249 (1.8)	2.58 (2.21, 3.00)	χ² = 79.5	<0.001*
Electrolyte Imbalance	1,242 (12.3)	741 (5.3)	2.34 (2.14, 2.55)	χ² = 254.8	<0.001*
Syncope/Collapse	297 (2.9)	563 (4.0)	0.74 (0.64, 0.85)	χ² = 10.8	<0.001*
Hypotension/Volume-Depletion Shock	270 (2.7)	172 (1.2)	2.19 (1.81, 2.65)	χ² = 47.0	<0.001*

Time-to-event analysis showed a significantly shorter time to any adverse event in the SCD cohort compared with the SCT cohort (log-rank p < 0.001) (Figure 2).

**Figure 2:**
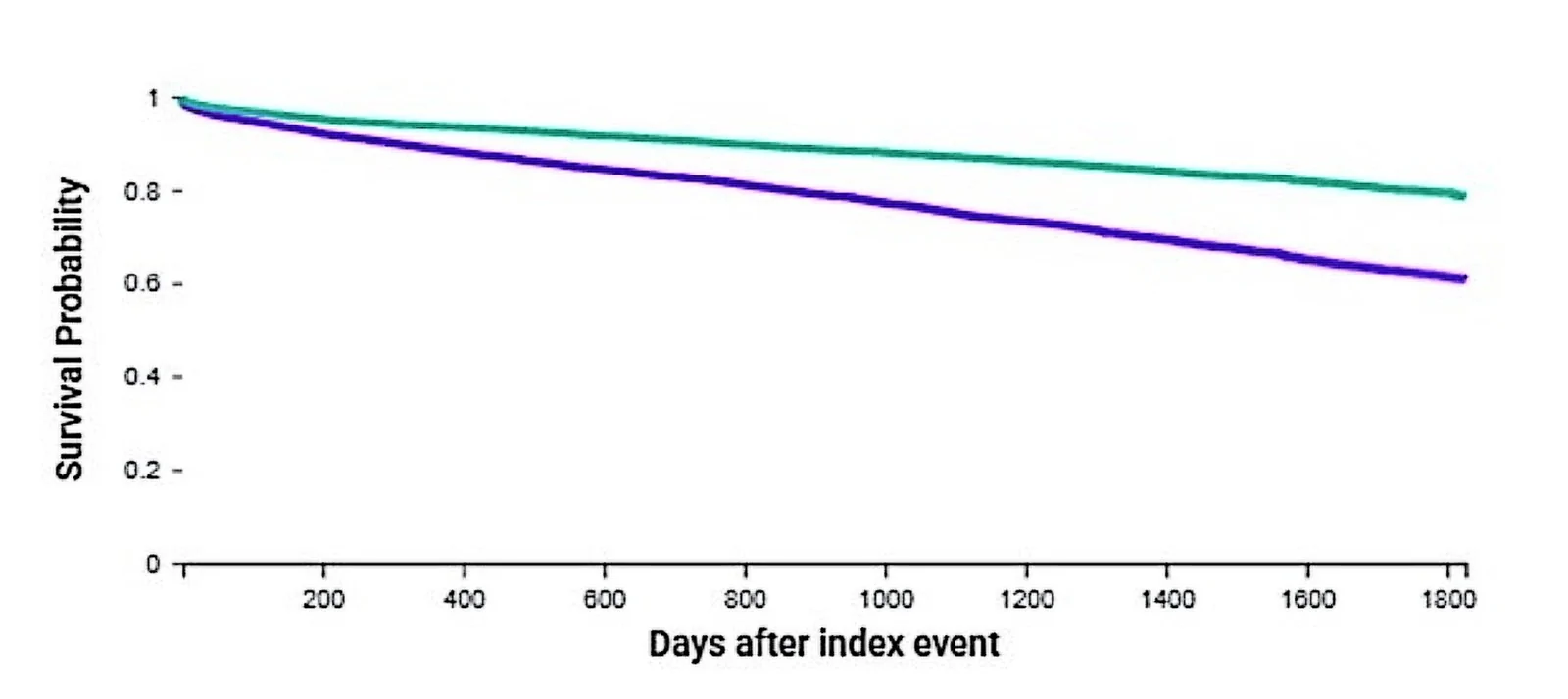
Subgroup Analysis: Kaplan-Meier Survival Curve Time to Adverse Event Comparing Sickle-Cell Disease to Sickle-Cell Trait Sickle-cell disease = purple line; Sickle-cell trait = green line; log-rank p < 0.001.

## Discussion

The findings of this study demonstrate that young adults with sickle-cell hemoglobinopathies carry a significantly higher burden of acute renal and hemodynamic complications compared to matched controls. Specifically, these patients exhibited a 62% increased risk of dehydration and a 76% increased risk of acute kidney failure. Furthermore, the cohort faced elevated risks for both electrolyte imbalances (RR = 1.66) and hypotension or volume-depletion shock (RR = 1.71). Although patients with SCD experienced higher absolute rates of most adverse outcomes, time-to-event analysis revealed that both SCD and SCT cohorts reached clinical endpoints significantly faster than the general population. A noteworthy finding was the higher frequency of syncope and collapse in the SCT group compared to the SCD cohort (4.0% vs. 2.9%). This suggests that while SCD may be more debilitating overall, SCT carriers possess a distinct vulnerability to acute hemodynamic collapse.

These results parallel established literature characterizing sickle hemoglobinopathies as definitive risk factors for acute physiological strain, particularly in high-performance environments [1,18]. Harmon et al. observed that NCAA Division 1 football players with SCT face a 37-fold higher relative risk of exertional death during high-intensity training compared to non-carriers [16]. This vulnerability is similarly documented in large military cohorts [7,8,10]. Nelson et al. conducted a prospective analysis of over 48,000 U.S. Army soldiers and noted a 54% increased adjusted risk of exertional rhabdomyolysis in SCT carriers [10]. Similarly, Webber et al. reported that while SCT carriers traditionally represent only 1.0% to 1.2% of the total U.S. Air Force population, they comprised 3.6% of those who experienced exertional rhabdomyolysis [7]. Longitudinal surveillance of NCAA SCT athletes from Wang et al. supports these clinical observations, noting altered baseline hematology, including significantly higher red cell distribution width (RDW), serum uric acid, and creatine kinase (CPK) levels across a competitive season [9]. The authors note that these elevated markers may serve as early subclinical indicators of muscle damage and kidney strain [9]. However, despite the elevated risk of specific exertional injuries, Nelson et al. observed that SCT was not associated with a higher overall risk of death among U.S. Army soldiers compared to individuals without the trait when universal training precautions were implemented [10]. Observations of severe outcomes associated with SCT status have prompted both the NCAA and the U.S. Army and other departments to implement guidelines and universal precautions regarding proper fluid balance and heat acclimatization to mitigate the risk of dehydration and sudden death [7-10,16]. While the combined SCD/SCT cohort in the present study showed higher rates of dehydration, acute kidney failure, electrolyte imbalance, and volume-depletion shock than matched controls, the SCT subgroup had a greater incidence of syncope/collapse than the SCD subgroup. These findings echo the exertional collapse documented among NCAA football players and military recruits [7-10,16].

The heightened risk of acute kidney injury (AKI) and dehydration in sickle-cell hemoglobinopathies is likely secondary to the early onset of sickle nephropathy [1,11,14,15,19]. This pathology is driven by the polymerization of HbS within the renal medulla, an environment characterized by low oxygen tension, acidosis, and hypertonicity that promote sickling [14,19]. Recurrent sickling induces progressive damage to the vasa recta, the specialized vascular system critical for maintaining the kidney’s osmolar gradient [1,19]. This disruption results in hyposthenuria, a diminished capacity to concentrate urine, a well-established complication of both SCD and SCT [14,20]. As hyposthenuria can lead to chronic water loss and depletion, individuals with sickle-cell hemoglobinopathies are predisposed to volume depletion and AKI when subjected to metabolic stressors such as hypoxia or heat [9,14]. Studies of NCAA SCT athletes have found that SCT players exhibit lower urine specific gravity and electrolyte concentrations before and after practice, as well as higher sweat sodium concentrations, suggesting altered kidney function and hydration status [9]. Consistent with these pathophysiological mechanisms, we observed that individuals with sickle-cell hemoglobinopathies experienced elevated risks of AKI, electrolyte imbalance, and dehydration compared to controls. Building on studies primarily focusing on SCT, our subgroup analysis further demonstrated a gradient of complication severity, with patients presenting with SCD having significantly increased risks of these acute events compared to SCT carriers.

Individuals with SCD and SCT have also been observed to typically exhibit significantly lower systolic, diastolic, and mean arterial blood pressures (BP) than race-, age-, and sex-matched norms from national datasets [15,20]. Consequently, blood pressure values considered optimal in the general population, such as a systolic BP of 120-139 mmHg or a diastolic BP of 70-89 mmHg, constitute “relative systemic hypertension” within this cohort [15,20]. A prospective study of 158 adults by Gordeuk et al. noted that the presence of relative systemic hypertension in adults with SCD was associated with an increased risk of pulmonary hypertension and renal dysfunction [15]. Furthermore, Pegelow et al. demonstrated that the risk for occlusive stroke increases significantly with systolic pressure, and mortality is higher for individuals whose pressures exceed the 90th percentile for the sickle-cell population [20]. These findings led the authors to suggest that initiating antihypertensive therapy at lower thresholds than those used in the general population might delay the progression toward pulmonary hypertension and renal failure [15]. These considerations align with our study’s observation of elevated risks for AKI and electrolyte imbalance in individuals with sickle-cell hemoglobinopathies compared to controls. However, this combined population also faced a 71% increased risk of hypotension or volume-depletion shock. This highlights a dichotomy clinicians face: how to effectively manage relative systemic hypertension to prevent long-term complications without inadvertently increasing vulnerability to acute hypotensive events [15,20].

Previous studies have noted that the effective management of SCD and SCT often involves a combination of disease-modifying therapies and structured supportive interventions [3]. For vaso-occlusive crises, Glassberg et al. emphasized aggressive pain control with intravenous opioids and patient-controlled analgesia and advised caution with excessive isotonic fluid resuscitation and potential pulmonary complications as a result [12]. Similarly, in a retrospective review of standardized order sets for adults with sickle cell, Parekh et al. reported that standardized admission order sets improved patient care metrics, including pain management, intravenous fluid management, and venous thromboembolism prophylaxis [13]. Despite the implementation of universal newborn screening, Ashorobi et al. and Creary et al. report that a significant proportion of SCT carriers remain unaware of their status [2,21]. This lack of awareness underscores the need for comprehensive education and the promotion of preventive behaviors and "universal precautions," such as standardized rehydration and monitored physical activity recovery, to mitigate potential complications [11,12]. These broad interventions could be beneficial because the primary symptoms of sickling, such as muscle cramping, weakness, and a dull ache, may often be misidentified as general heat exhaustion or cardiac arrhythmias [3,12,14]. To further enhance safety, Wang et al. have proposed that systematic medical surveillance using subclinical biomarkers, particularly elevated serum uric acid and CPK, might help identify individuals experiencing high physiological strain before a full clinical crisis develops [9]. The higher frequency of syncope and collapse observed in our SCT group compared to the SCD cohort highlights the need for additional research into the pathophysiology at play and into possible management options. There may be a need to integrate these universal precautions into routine emergency and inpatient management, but additional information is needed before developing recommendations. Collectively, these observations suggest that recognizing physiological vulnerability in carriers through broader clinical awareness and standardized care pathways could be of significant clinical value [1,3,11,12].

The results of this study must be considered in the context of its limitations. The exposure and outcomes were identified solely using ICD-10 diagnoses, which are subject to miscoding. Moreover, the database lacks detailed physiological and laboratory information, such as serial blood pressure measurements, renal function trends, or documented hydration and acclimatization protocols, which preclude assessment of disease severity or adherence to preventative strategies. Furthermore, over-the-counter medications, supplements, informal fluid-intake practices, and individual activity levels were not captured. The absence of activity-level data, particularly given our focus on the young-adult population rather than elite athletes or military personnel, is an important limitation that prevents assessment of physical exertion as a contributor. Additionally, despite propensity-score matching and multivariable adjustment, unmeasured confounders such as socioeconomic status, healthcare access, and comorbidities not coded likely remain. Finally, the follow-up period was limited to the duration of available records, restricting the evaluation of longer-term sequelae such as chronic kidney disease progression or pulmonary hypertension. These findings may not be generalizable to the broader SCD/SCT population, and additional prospective studies with detailed phenotyping, longer observation periods, and comprehensive clinical data are required to further inform sickle-cell management strategies in young adults.

## Conclusions

In this analysis, young adults with sickle-cell hemoglobinopathies demonstrated a markedly higher incidence of acute renal and hemodynamic events compared with propensity-matched controls. When examined by subgroup, individuals with SCT experienced syncope and collapse more frequently than those with SCD, highlighting a specific clinical vulnerability within the trait population. These findings have generated hypotheses that are worth prospective investigation, including the development of universal-precaution protocols and monitoring key subclinical biomarkers, which might help identify patients undergoing significant physiological strain before a clinical crisis develops. However, further prospective research incorporating detailed laboratory data and extended observation periods is necessary to determine if such strategies can be effectively translated into evidence-based care for the wider young-adult sickle-cell population.
